# Editorial: Microorganisms for Functional Food

**DOI:** 10.3389/fmicb.2016.00298

**Published:** 2016-03-08

**Authors:** Fabio Minervini, Maria De Angelis

**Affiliations:** Dipartimento di Scienze del Suolo, della Pianta e degli Alimenti, Università degli Studi di Bari Aldo MoroBari, Italy

**Keywords:** probiotic, fermentation, Lactobacilli, gut microbiome, health claim

Notwithstanding the European Food Safety Authority (EFSA) did not approve any health claims for probiotic foods [including terms such as “probiotic,” “active bacteria”; European Commission (EC), 2007], most of the research studies in the field of microorganisms used for obtaining functional food deal with (putatively) probiotic bacteria. Probiotics may positively affect human/animal health in several ways, such as by inhibiting gut pathogenic microorganisms (Delgado et al.), modulating immune response, lowering concentration of cholesterol in blood (Damodharan et al.), and exerting antioxidant activity. The mechanisms underlying the health effects of probiotic bacteria raise great interest among researchers. Modulation of the host gut microbiome represents an intriguing mechanism and is the subject of two research articles (Yang et al.; Senan et al.) published in this Research Topic. In detail, reutericyclin, a broad spectrum antimicrobial compound produced by *Lactobacillus reuteri* during feed fermentation, increased the abundance of *Dialister* and *Mitsuokella*, two *Firmicutes* genera that are gut commensals in weanling pigs (Yang et al.). Senan et al. reported that differences in gut microbiome composition (especially for *Lactobacillus, Clostridium, Eubacterium, Blautia, Shigella, Escherichia, Burkholderia*, and *Campylobacter*) affected the response of geriatric individuals to Lassi, a fermented milk containing a cholesterol-lowering strain of *Lactobacillus helveticus*.

Evaluation assays of probiotic potential are traditionally classified in two groups: “*in vitro*” and “*in vivo*.” The use of omics approaches is going to flank the traditional assays, thus probably speeding up research progress in the field of probiotics in the near future. For instance, sequencing of genomes will allow to rapidly detect and discard candidate probiotic microorganisms possessing genes coding for antibiotic resistance or virulence factors (Papadimitriou et al.). In this regard, an excellent example is provided in this Research Topic by Balzaretti et al. which performed comparative genome analysis of four strains of *Lactobacillus paracasei* in order to select the two best probiotic strains for oral usage. Anyway, *in vivo* approaches will keep on being unreplaceable, given the variability of host response to probiotics as affected by genotype, age, diet, variations in human environmental exposure, and composition of gut microbiome (Turnbaugh et al., 2009; Senan et al.).

Besides the understanding of the mechanisms underlying probiotic activities and studies dealing with interactions between probiotic microorganisms and gut microbiome, other future perspectives about microorganisms for functional food are: (i) novel putative probiotic bacteria, such as *Akkermansia muciniphila* and *Faecalibacterium prausnitzii* (Varankovich et al.); (ii) non-dairy food items (e.g., table olives) as carriers of probiotic bacteria (Rodríguez-Gómez et al.; Arroyo-López et al.); (iii) health effects of microbial metabolites (Yu et al.; Garrote et al.); and (iv) probiotic interventions on livestock indirectly benefiting human health (Yang et al.).

## Author contributions

FM resumed some of the contributions published in this research topic and wrote the Editorial. MD settled the structure of and reviewed the Editorial.

### Conflict of interest statement

The authors declare that the research was conducted in the absence of any commercial or financial relationships that could be construed as a potential conflict of interest.
